# Effect of the Menstrual Cycle on Electroencephalogram Alpha and Beta Bands During Motor Imagery and Action Observation

**DOI:** 10.3389/fnhum.2022.878887

**Published:** 2022-05-04

**Authors:** Rafaela Faustino Lacerda de Souza, Thatiane Maria Almeida Silveira Mendes, Luana Adalice Borges de Araujo Lima, Daniel Soares Brandão, Diego Andrés Laplagne, Maria Bernardete Cordeiro de Sousa

**Affiliations:** ^1^Behavioral Endocrinology Laboratory, Brain Institute, Federal University of Rio Grande do Norte, Natal, Brazil; ^2^Electroencephalography Laboratory, Brain Institute, Federal University of Rio Grande do Norte, Natal, Brazil; ^3^Behavioral Neurophysiology, Brain Institute, Federal University of Rio Grande do Norte, Natal, Brazil; ^4^Graduate Program in Psychobiology, Federal University of Rio Grande do Norte, Rio Grande do Norte, Brazil

**Keywords:** menstrual cycle, motor imagery, action observation, EEG, alpha-mu, beta-mu

## Abstract

Female sex steroids (FSS) can affect the motor system, modulating motor cortex excitability as well as performance in dexterity and coordination tasks. However, it has not yet been explored whether FSS affects the cognitive components of motor behavior. Mu is a sensorimotor rhythm observed by electroencephalography (EEG) in alpha (8–12 Hz) and beta (15–30 Hz) frequency bands in practices such as motor imagery (MI) and action observation (AO). This rhythm represents a window for studying the activity of neural circuits involved in motor cognition. Herein we investigated whether the alpha-mu and beta-mu power in the sensorimotor region (C3 and C4, hypothesis-driven approach) and the alpha and beta power over frontal, parietal, and occipital regions (data-driven approach) are modulated differently in the menstrual, follicular, and luteal phases of menstrual cycles in right-handed dominant women. To do so, these women underwent MI and AO in the three menstrual cycle phases. The spectral activity of the cortical regions for the alpha and beta bands were compared between phases of the menstrual cycle and a correlation analysis was also performed in relation to estrogen and progesterone levels. For the hypothesis-based approach, beta-mu event-related desynchronization (ERD) was significantly stronger in the C3 channel in the follicular phase than in the menstrual and luteal phases. For the data-driven approach, beta ERD during MI was higher in the follicular phase than in the menstrual and luteal phases in the frontal region. These findings suggest the effect of FSS on executive movement control. No effect of menstrual cycle phases was observed in cortical areas investigated during OA, but alpha and beta bands correlated positively with the follicular phase plasma estradiol level. Thus, the attenuation of alpha and beta bands referring to mirror neuron activities appears to be associated with inhibition of cortical activity when estradiol levels are lower, improving cognitive processing of motor action.

## Introduction

Female sex steroids (FSS—estradiol, progesterone, and their derivatives) exert wide-ranging influences on brain physiology and pathology. They induce plasticity in the primary sensorimotor cortex (Chen et al., 2009), modulate neural circuits involved in motor behavior (Arélin et al., 2015; Pritschet et al., 2020), and influence performance in tasks of fine manual dexterity and motor coordination (Hampson, 1990; Bayer and Hausmann, 2012; Mcewen et al., 2017). They are also associated with susceptibility to neurological diseases such as Parkinson’s Disease (PD), Stroke, Multiple Sclerosis and Amyotrophic Lateral Sclerosis, and the severity of the associated motor impairments (Quinn and Marsden, 1986; Czlonkowska et al., 2005).

FSS concentrations in women during reproductive age vary over an average period of 28 days, characterizing the menstrual cycle. This cycle can be divided into three phases considering the hormone concentration of FSS as follows: (1) menstrual phase, which corresponds to the menstruation period and is marked by low levels of circulating estradiol and progesterone; (2) follicular phase which is subsequent to the menstrual phase, in which estradiol levels begin to increase as a consequence of the increase in hypothalamic gonadotropins [follicle stimulating hormone (FSH) and luteinizing hormone (LH)], and whose outcome is ovulation; and (3) luteal phase immediately after ovulation in which FSS increases, mainly progesterone (Schuster et al., 2010; Fritz and Speroff, 2011; Poromaa and Gingnell, 2014).

The effect of FSS on the excitability of the primary motor cortex throughout the menstrual cycle in human studies has been investigated using transcranial magnetic stimulation (TMS) (Smith et al., 1999, 2002; Inghilleri et al., 2004; Rogers and Dhaher, 2017). These studies indicate that the phase of the menstrual cycle with high plasmatic progesterone levels (luteal) is associated with lower motor evoked potential (Smith et al., 1999, 2002), while the phase with high estradiol levels (late follicular) is associated with the highest motor evoked potential (Smith et al., 2002; Inghilleri et al., 2004). This suggests that progesterone and estradiol may have inhibitory and excitatory effects, respectively, on the corticospinal tract, although possible effects of FSS on non-nervous structures (e.g., muscles, tendons, and joints) cannot be ruled out. While FSS appears to affect tracts and mechanisms involved in motor control, their possible effects on motor cognition are unknown.

Motor imagery (MI), the act of imagining the movement without actually executing it, and motor action observation (AO) are practices capable of recruiting neural substrates involved in motor cognition (McFarland et al., 2000; Eaves et al., 2016; Angelini et al., 2018; Hardwick et al., 2018). However, MI integrates the activity of cortical and subcortical areas, while the AO only triggers the activity of cortical areas related to the mirror neuron system (Hardwick et al., 2018). MI is an important practice to improve motor performance when the repertoire of actions is simple (Jackson et al., 2003; Gatti et al., 2013), while AO seems to be a better strategy for learning more complex motor tasks (Gatti et al., 2013; Gonzalez-Rosa et al., 2015). In addition to being used for the rehabilitation of neurological damage and enhancing skilled performance, these practices are windows for the activity of neural circuits to investigate the processes involved in motor cognition (Moran and O’Shea, 2020).

Cortical activity during MI and AO, as well as movement execution, can be monitored using electroencephalography (EEG). All of these tasks decrease spectral power in the sensorimotor cortex, defining a rhythm called mu (Angelini et al., 2018; Bimbi et al., 2018; Festante et al., 2018; Montirosso et al., 2019; Jenson et al., 2020). This decrease occurs at two frequency bands, whose precise definitions vary between studies (Pfurtscheller and Aranibar, 1977; Hari and Salmelin, 1997; Muthukumaraswamy and Johnson, 2004; Pineda, 2005; Hari, 2006; Francuz and Zapała, 2011). One is alpha (referred to in this study as alpha-mu when located over the central regions), generally defined between 8 and 13 Hz (Pfurtscheller and Aranibar, 1977; Pineda, 2005; Francuz and Zapała, 2011; Angelini et al., 2018; Bimbi et al., 2018). The other is beta (referred to in this study as beta-mu when located over the central region), commonly occurring between 15 and 30 Hz (Klostermann et al., 2007; Bimbi et al., 2018). The decrease in power is referred to as event-related desynchronization (ERD), and is a correlate of the increased excitability in the thalamocortical system (Steriade and Llinás, 1988; Opri et al., 2019). On the other hand, the increase in power is referred to as event-related synchronization (ERS), and represents the maintenance of the *status quo* or inhibition of cortical activity (Steriade and Llinás, 1988; Hummel et al., 2002; Klimesch et al., 2006; Neuper et al., 2006; Engel and Fries, 2010).

The alpha-mu ERD induced by motor imagery is associated with increased motor evoked potential after single-pulse TMS (facilitatory stimulus) and with decreased intra-cortical inhibition after paired-pulse TMS with short interstimulus intervals (inhibitory stimulus) over the primary motor cortex (Takemi et al., 2013), which strengthens the idea of an association between alpha-mu ERD and motor cortex facilitation. Furthermore, alpha-mu ERD induced by motor imagery can be enhanced after transcranial anodal (excitatory) direct current stimulation (tDCS) over the primary motor cortex, and can be reduced after cathodic (inhibitory) stimulation (Matsumoto et al., 2010), suggesting an inverse relationship between alpha-mu amplitude and motor cortex excitability.

Beta ERD has been interpreted as a result of the coupling of several cortical (Lalo et al., 2007; Witham et al., 2007; Spinks et al., 2008; Kilavik et al., 2013) and subcortical (Levy et al., 2002a; Jenkinson and Brown, 2011; Opri et al., 2019) areas involved in sensorimotor integration. The coherence between the single-unit discharge in the somatosensory cortex and posterior parietal cortex and the local field potential of the primary motor cortex in the beta-band (Witham et al., 2007) is a suggestive example of cortical integration. Beta oscillations in the basal ganglia are modulated by voluntary movement and are coherent with the cortical beta rhythm (Kuhn et al., 2006; Klostermann et al., 2007; Jenkinson and Brown, 2011), which suggests the existence of cortical and subcortical region integration. Frontal regions participating in generating alpha and beta ERD/ERS during MI may indicate control of motor inhibition (Cebolla et al., 2017). FSS play an important role, especially estradiol, in modulating the basal nuclei activity, as demonstrated by the attenuation of motor symptoms associated with Parkinson’s disease (Quinn and Marsden, 1986).

The scientific literature presents evidence of the influence of FSS on the excitability of brain regions involved with motor behavior during rest, but limited to the complete study of motor control, leaving its effects on motor cognition unexplored. The hypothesis of this study is that cortical activity during cognitive motor processing varies depending on the menstrual cycle phases. Taking into account how cortical activity behaves when confronted with excitatory and inhibitory stimuli during cognitive motor processing and the modulatory effects of FSS in the cerebral cortex during rest, the aims of this study are: (I) to investigate whether alpha- and beta-mu rhythm varies during MI depending on the menstrual cycle phases; (II) to identify whether alpha and beta activity in other cortex areas related to visual processing, working memory, and inhibitory control during motor cognition tasks (MI and AO) vary depending on the menstrual cycle phases; (III) to investigate whether there are correlations between spectral power (alpha and beta) and FSS (estradiol, progesterone, and progesterone/estradiol ratio) in different menstrual cycle phases which indicates an association between FSS action and the cognitive motor response.

## Materials and Methods

### Participants

A total of 33 healthy young women (mean age ± standard deviation: 24.73 ± 2.74, range 18–30 years) participated in this study. They had no history of endocrine, neurological or psychiatric diseases. They were followed for 6 months to investigate the regularity of menstrual cycle length (between 22 and 35 days) (Creinin et al., 2004). The average cycle length was 28.52 ± 2.15 days. They had not taken hormone-based drugs for at least 6 months before this study. They were right-handed according to the Edinburgh Inventory (Oldfield, 1971; Brito et al., 1989), had normal hearing and normal or corrected-to-normal vision. Three participants were excluded from the study for the following reasons: one refused to participate in the second session due to incompatible time, one missed one of the EEG recording sessions, and the last one was excluded because her progesterone level was below 4 ng/mL in the luteal phase of the menstrual cycle.

The research project was submitted and approved by the ethics committee of the Federal University of Rio Grande do Norte, Rio Grande do Norte, Brazil (Comitê Central de Ética em Pesquisa—CEP Central/UFRN, approval number: 2.519.458). The project was also registered on the Brazilian clinical trials registry (*Registro Brasileiro de Ensaios Clínicos*—REBEC) with the code: RBR-422dc96 and the Universal Trial Number (UTN): U1111-1263-1222. All participants provided written consent before their participation in the research.

### Study Design

The participants underwent a familiarization session and three experimental sessions on different days. The familiarization session took place 1 week before the experimental sessions and each experimental session occurred in a different menstrual cycle phase (menstrual, follicular and luteal). The menstrual phase session occurred between days 2–5 of menses. The follicular phase session took place between days 9–12 of the menstrual cycle for cycles of 28 days. This interval and its duration varied proportionally according to the length of the participants’ cycles. The luteal phase session was performed 7–9 days after the LH surge. The LH was monitored in daily urine samples using LH tests (Famivita/Innovita—Tangshan Biological Technology Co., Ltd., Hebei, China; sensitivity: 20 mUI/ml; product format: strip). Nine participants had their first session in the menstrual phase, 11 participants in the follicular phase, and 10 participants in the luteal phase to diminish the order effect. The remaining sessions occurred during the two subsequent phases of the menstrual cycle.

The experimental conditions used in this study as well as the procedures for its application have been reproduced and adapted from a previous study (Eaves et al., 2016). During the familiarization session, the participants were introduced to MI practice and experimental conditions (see section “Familiarization Procedures”). None of the participants had previous knowledge about MI. A blood sample was collected at the beginning of each experimental session for hormonal dosage from each participant. They subsequently performed 5 different experimental paradigms in order: Finger-Tapping Test to investigation motor coordination; AO/MI protocols involved in motor cognition; Go/No Go Test dedicated to inhibitory motor control and movement execution; and, Hand Laterality Judgment Task to explore the motor component involved in the spatial transformation of body parts. This article was dedicated to investigating the effect of the menstrual cycle on motor cognition through the AO/MI protocols.

The participants in the MI condition watched a video of a female executant (instructed action) performing a rhythmical movement, and afterward imagined themselves performing the same action. The participants in the AO condition observed the image of an instructed rhythmical action, followed by a video of the executant performing the same action (see section “Experimental Conditions”). Unlike Eaves et al. (2016), the speed of instructed action was not manipulated and the participants did not verbally report (in AO) neither execute (in MI) the action in any trial. The cortical electrical activity of the participants was recorded using EEG during both conditions.

The instructed actions were to paint or wash the face using a bath sponge in the horizontal and vertical plane, totaling 4 types of stimuli (see section “Stimuli of the Action Observation and Motor Imagery Practices”). Each experimental block consisted of 8 repetitions of each stimulus chosen at random. Participants performed 4 blocks, 2 for each condition, in alternating order, totaling 64 trials per protocol. The condition for the first block was balanced between subjects and sessions following the rule: when the sum between the participant’s identification number and the experimental session number was even, the order was MI, AO, MI, AO, and when this sum was odd, the order was AO, MI, AO, MI. The interval between blocks was 2 min. Before the first block of each protocol, a training session with 8 trials was performed.

### Stimuli of the Action Observation and Motor Imagery Practices

**FIGURE 1:**
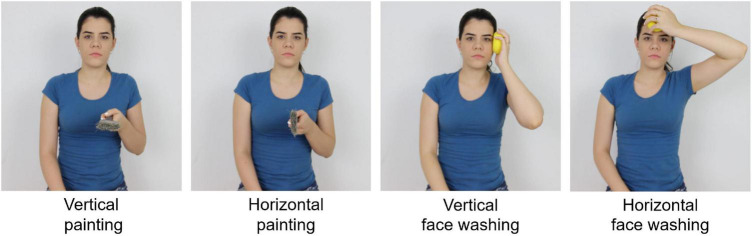
Instructed action stimuli types.

A Canon T3i digital camera was used to record the videos and photos of actions. The stimuli were created based on Eaves et al. (2016). They varied in terms of action type (painting and face washing) and the plane of motion (horizontal and vertical) to decrease the expectation for a given stimulus throughout the trials (Figure 1). The videos consisted of 2- and 4-s clips with one rhythmical action cycle per second (rhythmic speed: 60 bpm). The executant held a characteristic instrument in the photos to facilitate discrimination between instructed actions (Eaves et al., 2016). The 2-s video and the photos were used to instruct the action in the MI and AO conditions, respectively. The 4-s video was used to perform the AO condition.

The executant performed all actions in the photos and videos with the left hand generating mirrored images for the participants. This arrangement allows spatial compatibility which facilitates the action imitation when it was needed (e.g., Koski et al., 2003).

The stimuli were presented to participants on a 21.5-in LCD computer monitor (AOC, 2236VWA model) positioned 50 cm away from the head. The stimuli presentation was implemented using Psychopy (v 1.73.04) (Peirce, 2007, 2009).

### Familiarization Procedures

The familiarization session was divided into 6 steps. In step 1, the participants watched each of the four actions in the videos of the experimental conditions and imitated them (8 trials). They received verbal feedback on their motor performance. In step 2, they performed the motor actions while observing the corresponding action image for 4 s (8 trials). In step 3, they experienced with the AO condition trial framework but were asked to imagine themselves performing the action at the same time as the action video for 4 s (8 trials). In step 4, the participants were introduced to MI through a translated and adapted version of the Movement Imagery Questionnaire-3 (MIQ-3; from Williams et al., 2012). At the beginning, the participants performed the movements related to each of the three adapted actions of the MIQ-3 (to raise arm, to brush teeth and to bring a glass to the mouth) and then tried to feel themselves doing the movements without actually performing any movement (kinesthetic MI). Then, they reported the vivacity of each experience on a rating scale of 1–10, where 1 is “very difficult to feel” and 10 is “very easy to feel” (Williams et al., 2012). Finally, in step 5, they listened to a reading of a kinesthetic MI script with an action used in the experimental conditions. The participants followed the MI instructions with their eyes closed. In this last step (step 6), the participants were familiar with the procedures and practiced 1 block of each experimental condition (MI and AO).

### Experimental Conditions

Participants sat comfortably in a cushioned chair positioned in front of a computer monitor during the experimental conditions. Their right hand rested on a wooden board placed on their thighs and with the palm facing downwards. Their left hand was over a mouse also positioned on the wooden board.

**FIGURE 2:**
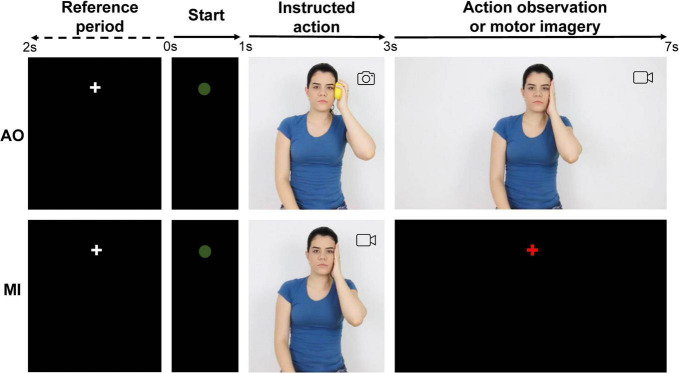
Sequence of events in the two experimental conditions. AO, action observation; MI, motor imagery.

The participants had to press the right mouse button with the left index finger to start each trial in both experimental conditions (based on Eaves et al., 2016). Then, a white fixation cross appeared on the screen for 1.5 s (baseline). Then it was replaced by a green circle for 1 s as a preparation cue (start). The participants in the AO condition were then shown an image with the instructed action for 2 s, followed by a video of the action observation for 4 s (Figure 2). They were instructed to always keep their gaze fixed on the executant’s right eye and focus attention on the observed action. After the green circle, the participants in the MI condition watched a video with the instructed action for 2 s, followed by a red fixation cross for 4 s. When staring at the red cross, the participants imagined themselves performing the instructed action with their right hand (motor imagery; Figure 2). Finally, the screen darkened in both conditions and the participant needed to click the mouse button again to start a new trial. The period of interest for EEG analysis corresponds to the period of action observation and motor imagery (3–7 s).

### Dosage of Female Sex Hormones

Blood samples were collected by venipuncture in a volume of 10 mL in vacutainer tubes between 8:00 and 9:00 a.m., preceding the beginning of the experimental tests. The samples were then centrifuged using 3,000 rpm for 10 min. The plasma was separated and then frozen and stored at –20°C. The samples were brought to room temperature on the day of the analysis and processed for dosages of estradiol and progesterone. Progesterone analyzes were performed using commercial kits from Immulite 2000^®^ (Catalog Number: L2KPW2, analytical sensitivity 0.1 ng/mL [0.3 nmol/L]; Siemens Healthcare Diagnostics, Germany) and Estradiol-17β analyzes with Access^®^ (kit Cat. no. B84493, Beckman-Coulter, Fullerton, CA, United States), both by chemiluminescent immunoassay. Intra-assay coefficient of variations was 8.36 + 8.27% for progesterone and 3.20 + 2.17% for estradiol, respectively.

### Electrophysiological Recording and Data Processing

EEG data were continuously recorded during the experimental conditions using a 64 channels actiCap electrode cap, BrainAmp DC amplifiers, and the BrainVision Recorder version 1.20.0506 software program (Brain Products, GmbH). The sampling rate was set to 1,000 Hz. The electrodes were positioned in caps of appropriately sized according to the 10–20 system and the electrical reference was located on the FCz. The electrode impedances were kept below 20 kΩ. Electromyography (EMG) data were also recorded on the same system with a sampling rate of 1,000 Hz. Surface Ag/AgCl electrodes were used over extensor digitorum communis muscles bilaterally and the reference electrode was positioned over the olecranon.

All data processing was performed using custom scripts written in MATLAB (version 8.5, 2015a, The MathWorks Inc., RRID:SCR_001622) and with the EEGLAB toolbox version 13.6.5b (RRID:SCR_007292) (Delorme and Makeig, 2004). The EEG data was filtered between 1 and 250 Hz using the EEGLAB basic FIR filter. Then, line noise was reduced using the CleanLine EEGLAB plugin. The bad channels (with high impedances or displacement during recording) were subsequently identified by investigating the joint normative probability of the average log power from 1 to 125 Hz in all channels (Gabard-Durnam et al., 2018). This assessment was carried out twice and the channels whose junction probability was greater than 3 standard deviations from the mean were rejected (mean ± standard deviation: 4.20 ± 2.6 channels). Independent component analysis (ICA) was performed as an artifact correction approach. Component rejection was automatized using ICLabel (Pion-Tonachini et al., 2019), which gives the probability that each component represents each of the 7 types of artifacts. Components with 80% confidence that represent the eye, muscle, or channel artifacts were rejected (21.3% of the components). Afterward, data was segmented at epochs with intervals between –1 and 7 s relative to the presentation of the green circle. Each channel at each epoch was further evaluated for the presence of artifact using the FASTER software program (Nolan et al., 2010; Gabard-Durnam et al., 2018). FASTER evaluates the variance of the channel in epoch, median gradient, amplitude range of the channel, and deviation from mean amplitude in the epoch for each channel to define z-scores; the bad channel is defined for the epoch when these z-scores are greater than 3 standard deviations from the mean. The bad channels for each epoch were then submitted to spherical interpolation (3.43 ± 0.68% of the channels by epoch). Thereafter, epochs including voltages above ± 100 μV were removed (10.96% of the trials). On average, 59.73 ± 0.53 trials of the AO protocol and 59.89 ± 0.82 trials of the MI protocol were used for analysis. Channels rejected before ICA were subjected to spherical interpolation, and finally the signal was referenced again to the average in all channels (average reference).

The time-frequency analysis was performed using the newtimef function of the EEGLAB toolbox (Delorme and Makeig, 2004). The signal epochs were subjected to a short-time Fourier Transform (STFT) over a 250 ms Hanning window sliding every 8 ms and in a frequency limit between 1 and 40 Hz to assess the event-related spectral perturbation (ERSP). Each time-frequency point value was divided by the average spectral power in the full-epoch length at the same frequency to reduce epoch noises (Ferree and Delorme, 2011). Thereafter, the time-frequency power was calculated by averaging the epochs. Finally, the spectral power estimated in each frequency was divided by the average power of the reference period (–1 to 0 s) and subjected to a logarithmic transformation to get the ERSP in order to visualize the power changes over the time and frequency range.

We evaluated the ERSP selecting electrodes by two different approaches. The first, a hypothesis-driven approach, comprised analyzes of alpha-/beta-mu rhythm on electrodes C3 and C4. The second, a data-driven approach, analyzed the average activity of ten electrode groups defined by the cortical regions: left prefrontal (Fp1, AF3, AF7), right prefrontal (Fp2, AF4, AF8), left frontal (F3, F5, F7, FC3, FC5), medial frontal (F1, Fz, F2, FC1, FCz, FC2), right frontal (F4, F6, F8, FC4, FC6), medial central (C1, Cz, C2, CP1, CPz, CP2), left parietal (P3, P5, P7, PO3, PO7), medial parietal (P1, Pz, P2, POz), right parietal (P4, P6, P8, PO4, PO8) and occipital (O1, Oz, O2). The regions of interest and channels were selected considering the EEG regions (pre-frontal, frontal, central, parietal, occipital), and the topographic characterization associated with the MI and AO protocols (medial and lateral components). There are lateral components (alpha and beta-ERD) in MI protocol and there is medial component (alpha and beta-ERS) in AO.

We chose to use only two electrodes for the hypothesis-based analysis for two reasons. First, according to preliminary analyses, these two electrodes (C3 and C4) showed a robust response for identification of ERS/ERD associated with motor imagery (for alpha and beta bands) and observation of action (for beta band) and therefore, to verify if there is an effect of the menstrual cycle on the cognitive processing of these tasks. Second, other authors observed modulation in cortical activity at these same two electrodes during motor tests such as MI and AO (Pfurtscheller et al., 2006; Neuper et al., 2009; Babiloni et al., 2014) and during motor diseases (Bizovicˇar et al., 2014).

The average spectral power between 3.15 and 7 s (Figure 2) was computed for alpha (8–13 Hz) and beta band (15–30 Hz) for each cortical region and experimental condition. The decrease in ERSP below the baseline was interpreted as an increase in ERD. The increase in ERSP above the baseline was interpreted as an increase in ERS.

EMG data was filtered between 10 and 499 Hz using the EEGLAB basic FIR filter, rectified and divided into epochs containing two-time windows: reference period of 2 s (cross period) and MI/AO period of 4 s. Root mean square (RMS) values of the EMG signal was calculated for each epoch and then averaged per subject for both time windows to investigate the background EMG activity.

### Statistical Analysis

Statistical tests were conducted using MATLAB software (version 8.5, 2015a, The MathWorks Inc.). Part of the data were not normally distributed according to Kolmogorov-Smirnov test, so it was decided to use non-parametric tests. One-Sample Wilcoxon Signed Rank test compared to zero was conducted to determine whether alpha and beta power changed significantly relative to the baseline period during the experimental conditions (MI and OA). The Friedman test was performed to compare the difference in mean ranks response between menstrual cycle phases for alpha and beta power for each experimental condition. Statistical significance was assessed by the Tukey–Kramer *post-hoc* test. All analyzes were applied to the ERSP of the electrodes C3 and C4 to specifically investigate the influence of hormonal changes on the mu rhythm (alpha-mu and beta-mu) on the sensorimotor cortex for a hypothesis-driven analyzes, and they were applied to the other cortical regions with *p*-value corrected for multiple comparisons on a data-driven approach. *P*-values were corrected by the number of cortical regions using false discovery rate (FDR) (Benjamini et al., 1995).

In order to ensure that background EMG activity was not enhanced during MI/AO practice, we analyzed if there was a difference for RMS mean between two-time windows: reference period and MI/AO time course using Two-Sample Wilcoxon Signed Rank test for each menstrual cycle phase.

The results were presented in spectrograms, topographies, boxplots, and scatterplots overlain with average power values per subject. The effect sizes were reported as correlation coefficients r for Wilcoxon Signed Rank Tests and Kendall’s W for Friedman tests. Spearman’s correlation was used to verify the relation between independent variables and hormone levels in the three different menstrual cycle phases. *P*-values < 0.05 were considered to be indicative of statistical significance.

## Results

### Female Sex Steroids Across the Menstrual Cycle

**FIGURE 3:**
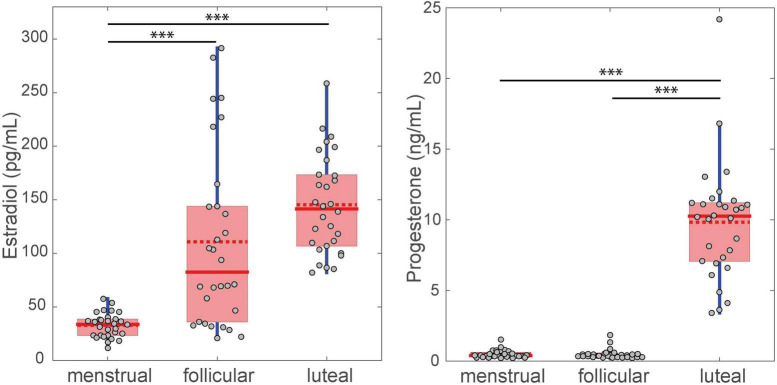
Difference between FSS levels for the three menstrual cycle phases. The filled red line in the boxplot represents the median and the dotted red line represents the mean. ^∗∗∗^*p* < 0.001.

The plasma concentrations of estradiol and progesterone in each phase of the menstrual cycle are shown in Figure 3. The phase effect was significant for estradiol [χ^2^(2) = 41.27, *p* < 0.001, *W* = 0.69] and progesterone [χ^2^(2) = 45.82, *p* < 0.001, *W* = 0.77] levels. *Post-hoc* testing showed that the estradiol level significantly increased in the follicular (mean ± standard deviation = 110.67 ± 82.49 pg/mL) and luteal phases (145.33 ± 46.01 pg/mL) compared to the menstrual phase (32.86 ± 11.24 pg/mL, *p* < 0.001). No difference was found for estradiol between the follicular and luteal phases (*p* = 0.084). Progesterone levels significantly increased in the luteal phase (9.81 ng/mL ± 4.11) compared to the menstrual (0.48 ± 0.27 ng/mL, *p* < 0.001) and follicular phases (0.47 ± 0.34 ng/mL, *p* < 0.001). No difference was observed between the menstrual and follicular phases (*p* = 1) for progesterone concentration.

### Background Electromyography Activity

No significant differences were found between MI/AO and reference period in different phases of the menstrual cycle for the average RMS of the right [AO: menstrual (*z* = 4.24, *p* = 1, *r* = 0.37), follicular (*z* = 3.15, *p* = 1, *r* = 0.28), luteal (*z* = 2.38, *p* = 1, *r* = 0.21); MI: menstrual (*z* = –0.47, *p* = 0.32, *r* = 0.04), follicular (*z* = 2.18, *p* = 0.98, *r* = 0.19), luteal (*z* = 2.16, *p* = 0.98, *r* = 0.19)] and left arm [AO: menstrual (*z* = 4.22, *p* = 1, *r* = 0.37), follicular (*z* = 3.74, *p* = 1, *r* = 0.33), luteal (*z* = 4.17, *p* = 1, *r* = 0.36); MI: menstrual (*z* = 4.24, *p* = 1, *r* = 0.37), follicular (*z* = 4.67, *p* = 1, *r* = 0.41), luteal (*z* = 3.78, *p* = 1, *r* = 0.33)].

### Motor Imagery

**FIGURE 4:**
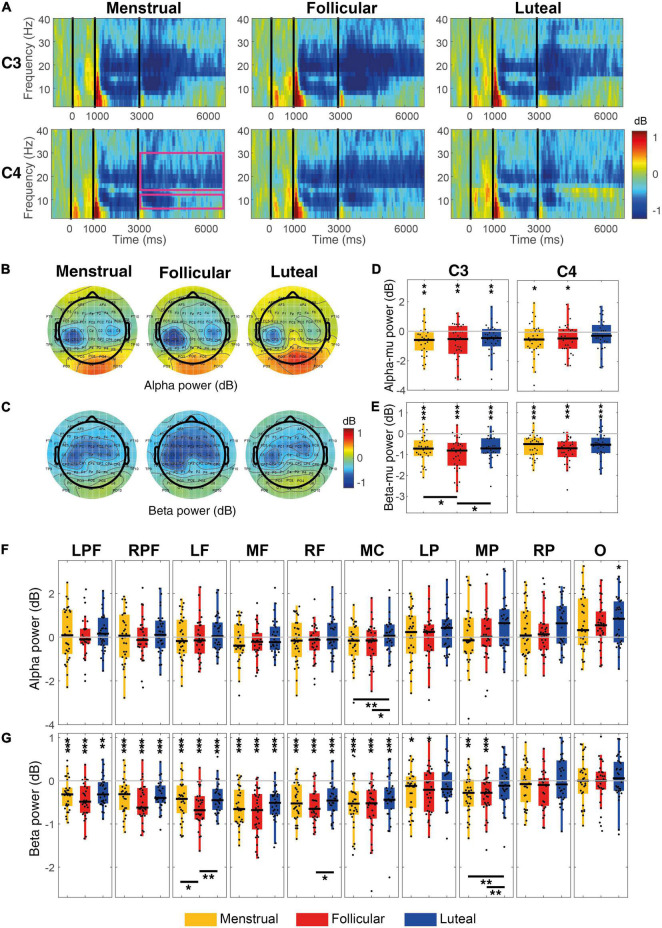
Event-related power for each menstrual cycle phase during the MI condition. **(A)** Displays time-frequency plots of C3 and C4 electrodes by menstrual cycle phase. The pink rectangles in the first column and C4 row indicate the time-frequency interest limits: Event B time (3.15–7 s), alpha-band (8–13 Hz), and beta-band (15–30 Hz). **(B)** Shows topographies of the mean alpha power for each menstrual cycle phase and **(C)** shows topographies of the mean beta power for each menstrual cycle phase. Boxplots and scatter plots are presented in **(D)** with alpha-mu power, in **(E)** with beta-mu power per menstrual cycle phase and per cortical region, in **(F)** with alpha power for various regions of the cortex, and in **(G)** with beta power for various cortex regions. Asterisks located above boxplots indicate that the power was significantly different from zero in the menstrual cycle phase below. Asterisks located below boxplots indicate that the power was significantly different between the two menstrual cycle phases indicated by the horizontal line. *p < 0.05, **p < 0.01, ***p < 0.001; C3, left central; C4,: right central; LPF, left prefrontal, RPF, right prefrontal, RF, right frontal, MF, medial frontal, LF, left frontal, MC, medial central, LP, parietal, MP, medial parietal, RP, right parietal and O, occipital.

Alpha-mu ERD during MI increased significantly when compared to zero in the left hemisphere (C3) for the menstrual (mean ± standard deviation of alpha power = –0.64 dB ± 0.18, *z* = –3.18, *p* = 0.0015, *r* = 0.41), follicular (–0.77 dB ± 0.24, *z* = –2.68, *p* = 0.0073, *r* = 0.35) and luteal (–0.54 dB ± 0.18, *z* = –2.78, *p* = 0.0053, *r* = 0.36) phases (Figures 4A,B,D). Alpha-mu ERD in the right hemisphere (C4) significantly increased for both menstrual (–0.54 dB ± 0.22, *z* = –2.31, *p* = 0.02, *r* = 0.30) and follicular phases (–0.60 dB ± 0.25, *z* = –2.11, *p* = 0.035, *r* = 0.27), but it was not significant for the luteal phase (–0.21 dB ± 0.19, *z* = –0.96, *p* = 0.33, *r* = 0.12) (Figures 4A,B,D). When considering the other cortex regions, only the occipital region presented a significant increase of the alpha ERS, and only for the follicular phase (–0.57 dB ± 0.18, *z* = –2.91, *p* = 0.036, *FDR corrected, r* = 0.38) (Figures 4B,F).

Using the hypothesis-driven approach, no significant difference was observed between the menstrual cycle phases for alpha-mu rhythm [C3: *χ^2^*(2) = 2.60, *p* = 0.27, *W* = 0.04; C4: *χ^2^*(2) = 1.27, *p* = 0.53, *W* = 0.02] (Figures 4A,B,D). However, using the data-driven approach, a difference between phases was observed in the medial central region [*χ^2^*(2) = 11.67, *p* = 0.003, *FDR corrected*, *W* = 0.19], where alpha ERSP was greater in the luteal phase than in the menstrual (*p* = 0.004) and follicular (*p* = 0.027) phases (Figures 4B,F).

Beta-mu ERD was significantly strong during MI when compared to zero in both hemispheres in menstrual (C3: –0.74 dB ± 0.12, *z* = –4.25, *p* < 0.001, *r* = 0.55; C4: –0.62 dB ± 0.09, *z* = –4.39, *p* < 0.001, *r* = 0.57), follicular (C3: –0.99 dB ± 0.15, *z* = –4.61, *p* < 0.001, *r* = 0.60; C4: –0.80 dB ± 0.11, *z* = –4.72, *p* < 0.001, *r* = 0.61) and luteal phases (C3: –0.69 dB ± 0.11, *z* = –4.43, *p* < 0.001, *r* = 0.57; C4: –0.55 dB ± 0.11, *z* = –3.92, *p* < 0.001, *r* = 0.51) (Figures 4A,C,E). When compared to zero, beta ERD during MI in all prefrontal and frontal, and medial central regions was significantly stronger for all three phases (*all ps* ≤ 0.01, *FDR corrected*) and in left and medial parietal regions it was significantly stronger for menstrual and follicular phases (*all ps* ≤ 0.02, *FDR corrected*) (Figures 4C,G).

Even in the hypothesis-driven approach, beta-mu ERD significantly varies between the menstrual cycle phases in C3 [*χ^2^*(2) = 8.47; *p* = 0.014; *W* = 0.14], but not in C4 [*χ^2^*(2) = 2.47; *p* = 0.29; *W* = 0.04]. Beta-mu ERD in the left hemisphere (C3) was stronger in follicular phase than in the menstrual (*p* = 0.037) and luteal (*p* = 0.027) phases (Figures 4A,C,E). The menstrual cycle phases in the data-driven approach were significantly different for the beta ERD in the left frontal [*χ^2^*(2) = 11.40; *p* = 0.017, *FDR corrected*; *W* = 0.19], right frontal [*χ^2^*(2) = 8.60; *p* = 0.045, *FDR corrected*, *W* = 0.14] and medial parietal [*χ^2^*(2) = 12.87; *p* = 0.016, *FDR corrected*, *W* = 0.21] regions during the MI condition. Beta ERD was significantly stronger in the follicular phase than in the menstrual (*p* = 0.018) and luteal (*p* = 0.005) phases for the left frontal region and in relation to the luteal phase (*p* ≤ 0.018) for the right frontal region. Beta-ERD in the medial parietal region was weaker in the luteal phase when compared to the menstrual (*p* = 0.008) and follicular (*p* = 0.004) phases (Figures 4C,G).

No correlation was observed between the alpha amplitude generated during MI and the estradiol and progesterone levels and the progesterone/estradiol ratio for any of the cortical regions (*all ps* > 0.05). Likewise, no correlation was observed between the beta amplitude and hormone levels (*all ps* > 0.05).

The Supplementary Material present tables with more statistical details for MI analysis.

### Action Observation

**FIGURE 5:**
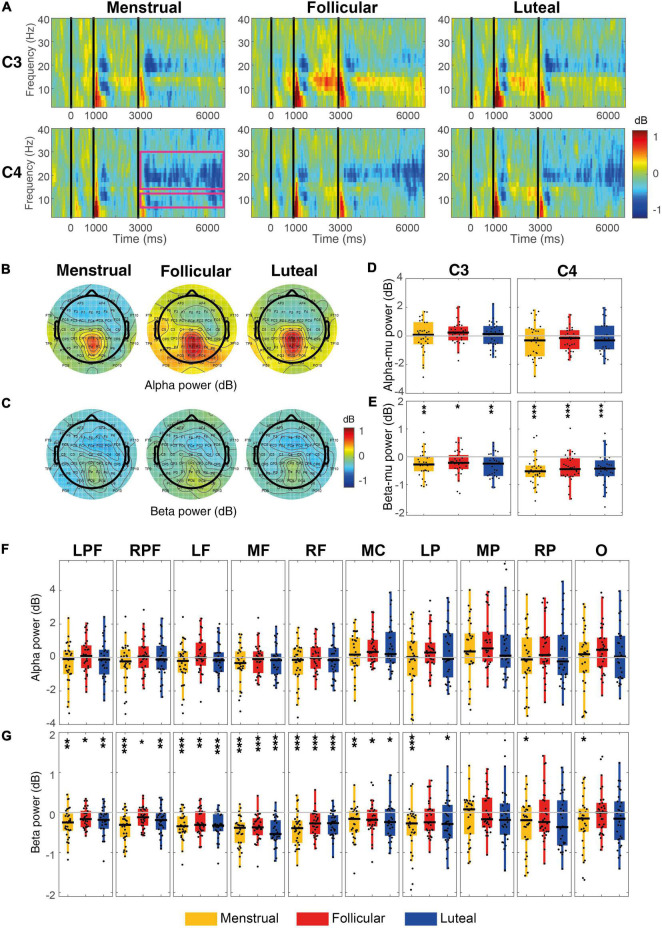
Event-related power for each menstrual cycle phase during the AO condition. **(A)** Displays time-frequency plots of C3 and C4 by menstrual cycle phase. The pink rectangles in the first column of the C4 row indicate the time-frequency interest limits: Event B time (3.15–7 s), alpha-band (8–13 Hz), and beta-band (15–30 Hz). **(B)** Shows topographies of the mean alpha power for each menstrual cycle phase. **(C)** Shows topographies of the mean beta power for each menstrual cycle phase. Boxplots and scatter plots are presented in **(D)** with alpha-mu power, in **(E)** with beta-mu power per phase of the menstrual cycle and per cortical region, in **(F)** with alpha power for various cortex regions, and in **(G)** with beta power for various cortex regions. Asterisks located above boxplots indicate that the power was significantly different from zero in the menstrual cycle phase below. Asterisks located below boxplots indicate that the power was significantly different between the two menstrual cycle phases indicated by the horizontal line. *p < 0.05, **p < 0.01, ***p < 0.001; C3, left central, C4, right central, LPF, left prefrontal, RPF, right prefrontal, RF, right frontal, MF, medial frontal, LF, left frontal, MC, medial central, LP, parietal, MP, medial parietal, RP, right parietal and O, occipital.

No significant increase in alpha-mu ERD was observed for the AO condition in the menstrual (C3: –0.04 dB ± 0.21, *z* = 0.07, *p* = 0.94, *r* = 0.009; C4: –0.36 dB ± 0.22, *z* = –1.34, *p* = 0.17, *r* = 0.17), follicular (C3: –0.22 dB ± 0.14, *z* = –1.66, *p* = 0.09, *r* = 0.21; C4: –0.25 dB ± 0.15, *z* = –1.75, *p* = 0.07, *r* = 0.23) or luteal phases (C3: 0.05 dB ± 0.16, *z* = 0.19, *p* = 0.84, *r* = 0.02; C4: –0.21 dB ± 0.19, *z* = –1.45, *p* = 0.14, *r* = 0.18) (Figures 5A,B,D). Alpha ERSP also did not present significant changes in any cortical region (*all ps* ≥ 0.05; Figures 5B,F) during AO. Although the medial central and medial parietal regions showed an apparent increase of the ERS in the three phases of the menstrual cycle during this condition, only a trend to statistical significance was found for the follicular phase (medial central: 0.53 dB ± 0.17, *z* = 2.54, *p* = 0.055, *FDR corrected, r* = 0.33; medial parietal: 0.92 dB ± 0.28, *z* = 2.72, *p* = 0.055, *FDR corrected, r* = 0.35; Figures 5B,D).

Beta-mu ERD during AO increased significantly compared to zero for the menstrual (C3: –0.27 dB ± 0.08, *z* = –3.05, *p* = 0.002, *r* = 0.39; C4: –0.50 dB ± 0.09, *z* = –3.75, *p* < 0.001, *r* = 0.49), follicular (C3: –0.22 dB ± 0.08, *z* = –2.36, *p* = 0.019, *r* = 0.30; C4: –0.41 dB ± 0.09, *z* = –3.67, *p* < 0.001, *r* = 0.47), and luteal phases (C3: –0.31 dB ± 0.07, *z* = –3.16, *p* = 0.007, *r* = 0.41; C4: –0.45 dB ± 0.10, *z* = –3.65, *p* < 0.001, *r* = 0.47) (Figures 5A,C,E). This was also observed for all prefrontal (*all ps* ≤ 0.03, *FDR corrected*), frontal (*all ps* ≤ 0.001, *FDR corrected*) and medial central (*all ps* ≤ 0.03, *FDR corrected*) regions in the three menstrual cycle phases. There was an increase in the menstrual (–0.41 dB ± 0.08, *z* = –3.63, *p* < 0.001, *r* = 0.47) and luteal (–0.24 dB ± 0.10, *z* = –2.23, *p* = 0.03, *FDR corrected*, *r* = 0.29) phases for the left parietal region, while the beta ERD increase for the right parietal regions only occurred in the menstrual phase (–0.29 dB ± 0.11, *z* = –2.21, *p* = 0.03, *FDR corrected, r* = 0.28) (Figures 5C,G).

No significant difference between menstrual cycle phases was found for alpha-mu [C3: *χ^2^*(2) = 1.87; *p* = 0.39, *W* = 0.03; C4: *χ^2^*(2) = 0.80; *p* = 0.67, *W* = 0.01; Figures 5A,B,D,F], or beta-mu [C3: *χ^2^*(2) = 1.40; *p* = 0.50, *W* = 0.02; C4: *χ^2^*(2) = 1.27; *p* = 0.53, *W* = 0.02; Figures 5A,C,E,G; hypothesis-driven approach], nor for alpha or beta power bands in any cortical region during the AO condition (*ps* ≥ 0.05; Figures 5C,G; data-driven approach).

**FIGURE 6:**
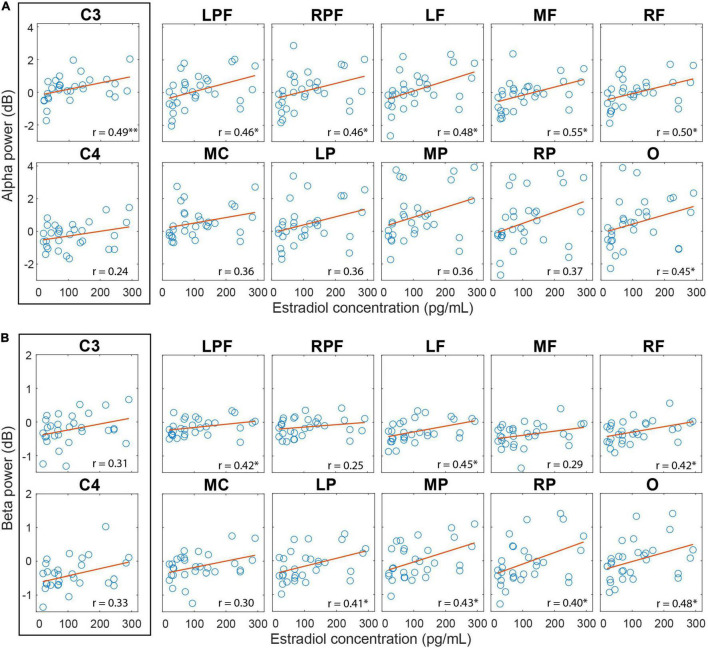
Correlation between cortical activity during AO and estradiol levels in the follicular phase. **(A)** Correlation between the alpha amplitude and the estradiol level. **(B)** Correlation between beta amplitude and estradiol level. Regions with significant correlations have an asterisk. *P*-value: < 0.001 (^∗∗^), < 0.05 (^∗^); FDR correction.

The correlation analysis between the alpha amplitude during AO and the estradiol and progesterone levels and the progesterone/estradiol ratio revealed a moderate positive correlation between estradiol levels in the follicular phase and the alpha amplitude during AO for the C3 electrode (*rho* = 0.49; *p* = 0.01), left prefrontal (*rho* = 0.46; *p* = 0.02, *FDR corrected*), right prefrontal (*rho* = 0.46; *p* = 0.02, *FDR corrected*), left frontal (*rho* = 0.48; *p* = 0.02, *FDR corrected*), frontal medial (*rho* = 0.56; *p-FDR* = 0.02), frontal right (*rho* = 0.50; *p* = 0.02, *FDR corrected*) and occipital (*rho* = 0.45; *p* = 0.02, *FDR corrected*) regions (Figure 6A).

The correlation between beta amplitude during AO and estradiol levels in follicular phase was also significant and positive for the left prefrontal (*rho* = 0.42; *p* = 0.04, *FDR corrected*), left frontal (*rho* = 0.45; *p* = 0.04, *FDR corrected*), right frontal (*rho* = 0.42; *p* = 0.04, *FDR corrected*), central left (*rho* = 0.48; *p* = 0.04, *FDR corrected*), left parietal (*rho* = 0.42; *p* = 0.04, *FDR corrected*), medial parietal (*rho* = 0.43; *p* = 0.04, *FDR corrected*), right parietal (*rho* = 0.40; *p* = 0.04, *FDR corrected*) and occipital (*rho* = 0.48; *p* = 0.04, *FDR corrected*) regions (Figure 6B). No correlation with progesterone levels or with the progesterone/estradiol ratio was found.

The Supplementary Material present tables with more details of the AO analysis.

## Discussion

Alpha and beta ERD are electroencephalographic correlates found in the cerebral cortex during MI, AO, or execution of a motor action. Their magnitudes vary depending on the excitatory or inhibitory inputs in the motor cortex that arrive from other cortical and subcortical areas. Considering the potential modulatory effect of the FSS in the sensorimotor and frontal motor processing areas, this study investigated whether cortical activity in alpha and beta bands varies depending on menstrual cycle phases during the practice of MI and AO.

EMG analysis did not detect significant differences that would indicate execution of voluntary arm movement during MI and AO practices as expected. In our study, plasma FSS levels showed a fluctuation pattern compatible with regular menstrual cycles in all participants. The menstrual phase is marked by low levels of the FSS (estrogen and progesterone) under normal conditions due to the negative feedback from the hypothalamic GnRH hormone and the hypophyseal LH and FSH gonadotropins with their production in the ovaries. Estradiol levels increase in the follicular phase, reaching their highest levels about 24 h before ovulation, around the middle of the cycle. Then, estrogen and, more markedly, progesterone secretion begin after ovulation, initiating the luteal phase which is characterized by high levels of progesterone l and also elevated estradiol (Schuster et al., 2010; Fritz and Speroff, 2011; Poromaa and Gingnell, 2014).

We show that alpha-mu ERD in the left motor cortex (C3) is significantly stronger during MI of the right arm compared to zero for all phases of the investigated menstrual cycle and in the right motor cortex (C4) for the menstrual and follicular phases. The engagement of the contralateral hemisphere in performing a lateralized imagined action is an indication that similar mechanisms are involved in motor imagery and movement execution, and has been reported in several studies (McFarland et al., 2000; Eaves et al., 2016; Angelini et al., 2018; Hardwick et al., 2018). Additionally, we showed that beta-mu ERD was significantly stronger during MI compared to zero in central regions (C3 and C4), bilaterally, in all menstrual cycle phases. The same result was also observed for beta power in the prefrontal, frontal, and medial central regions. This effect was also observed in medial and left parietal regions in the menstrual and follicular phases. This result is in line with the literature that shows although beta ERD is stronger in the contralateral hemisphere, both hemispheres present beta ERD during MI (Nakagawa et al., 2011; Kilavik et al., 2013; Kraeutner et al., 2014; Eaves et al., 2016). Beta ERD also has a generalized distribution when compared to alpha ERD (McFarland et al., 2000).

Beta-mu ERD was higher in the follicular phase than other phases during MI and no significant difference was found between menstrual cycle phases for alpha-mu ERD.

Studies with source localization report that alpha-mu, located in the hand area which extends to the postcentral cortex, reflects the function of the somatosensory cortex, and beta-mu, located in the precentral gyrus, is associated with the function of the motor cortex (Salmelin et al., 1995; Hari and Salmelin, 1997; Ritter et al., 2009). Added to this evidence, our findings suggest that high levels of estradiol in the follicular phase can consistently influence the neural circuits more specifically involved with motor processing. This result corroborates evidence that indicates greater facilitation of the corticospinal tract at this menstrual cycle phase (Matsumoto et al., 2010).

The alpha ERSP in the central medial region was not significantly different from zero. Nonetheless, alpha ERSP was positively stronger in the luteal phase than other phases in the medial central region. According to the “focal ERD/surround ERS” theory, ERD in motor regions is associated with the thalamo-cortical circuit which facilitates focal activation of this cortical region, while the surrounding ERS is a consequence of the thalamo-cortical inhibitory modulation in other regions (Suffczynski et al., 2001; Neuper et al., 2006). Thus, once the representation area of the hands (C3 and C4) is being activated, the representation area of the feet (central medial region) is inhibited. The highest alpha ERSP recorded in the medial central region in the luteal phase may be associated with the potentiation of this inhibitory effect, considering the already known inhibitory effect of progesterone on the motor cortex (Smith et al., 1999, 2002).

Beta ERD during MI was significantly stronger in the right and left frontal regions in the follicular phase than in the menstrual and luteal phases. Beta activity in the frontal region and outside the sensorimotor cortex may represent executive control of the movement (Brown and Williams, 2005; Swann et al., 2009; Wessel et al., 2013), probably through communication between basal ganglia and frontal regions (Schmidt et al., 2019). This result adds important contributions to the motor-cognitive model (Glover and Baran, 2017; Glover et al., 2020). According to this model, motor imagery involves frontal executive processes that go beyond those observed during the planning and execution of motor action (Van der Lubbe et al., 2021). This extra activity would be associated with inhibitory control that allows movement retention during MI (Angelini et al., 2015, 2016). Thus, our findings in the frontal region suggest that female sex hormones may be modulating the executive control of the MI as well as of the AO.

An important point to note is that PD is associated with increased beta power in the basal ganglia and cortex (Levy et al., 2002b; Williams et al., 2002; Sharott et al., 2005; Kuhn et al., 2006; Hammond et al., 2007; Alavi et al., 2013) and treatments with levodopa or deep brain stimulation contribute to restoring physiological beta activity (Silberstein et al., 2005; Hammond et al., 2007). Considering the evidence of the influence of estradiol on the nigrostriatal system and its implications on PD (Varshney et al., 2000; Lee et al., 2019), the increase in beta-ERD on the frontal region during the follicular phase of the menstrual cycle may be an indication of facilitated cholinergic pathways between basal ganglia and the cortex of this region.

We also observed that beta ERD in the medial parietal regions was weaker in the luteal phase than in other phases. The activity of the parietal region during MI practice is frequently associated with task vividness (Sirigu et al., 1996; Lorey et al., 2011; Lyu et al., 2016; Zabicki et al., 2019). A study shows that beta-ERD coherence between the motor and parietal cortex is related to precise motor performance (Chung et al., 2016). The attenuation of beta-ERD in the medial parietal cortex observed during MI may indicate less MI vividness and weaker communication between these areas in the luteal phase of the menstrual cycle, but unfortunately, we were not able to measure this variable.

Contrary to what was expected, it was not possible to observe significant alpha-mu ERD during AO in this study. This result is unexpected for being in disagreement with studies that describe strong alpha-mu ERD similar to performing movement and MI during AO (Eaves et al., 2016; Angelini et al., 2018; Montirosso et al., 2019). This result may be associated with the fact that the AO perspective influences alpha-mu activity (Angelini et al., 2018). When AO is performed in the third-person perspective, as in this study, the alpha-mu reactivity tends to be lower than in the first-person perspective. However, there is no difference between these perspectives for beta-mu. In this study, beta ERD during AO was significantly stronger against zero in all prefrontal, frontal and central regions (beta-mu) in all menstrual cycle phases and, with less evidence, in the parietal and occipital regions, as observed in MI. This result is supported by studies which shows that the beta ERD during MI and AO is more diffuse with peak bias in the vertex (McFarland et al., 2000; Schaller et al., 2017). The beta ERD similarity between the protocols can be explained by the fact that both tasks may be activating neural circuits involved in motor cognition (Buccino et al., 2001; Caspers et al., 2010; Eaves et al., 2016; Hardwick et al., 2018). However, there was no significant difference between menstrual cycle phases for alpha and beta ERD in any cortical region during AO practice. Unlike MI practice, which recruits cortical and subcortical areas, AO does not appear to substantially recruit the activity of subcortical areas (Hardwick et al., 2018), nullifying the possible effects of FSS, especially the facilitatory effect of estradiol on communication between basal ganglia and frontal cortex.

Although the follicular phase of the menstrual cycle presents a stronger beta-mu ERD in the C3, bilateral frontal region, while the luteal phase presents a weaker beta-ERD in the parietal medial region during MI, it was not possible to evidence an association between the serum hormonal levels (estradiol and progesterone in the follicular and luteal phases, respectively) and the amplitude of the beta ERD during MI. Interestingly, the estradiol level in the follicular phase and spectral power (alpha and beta) showed moderate positive correlations in several cortical regions during AO. The estradiol levels and progesterone/estradiol ratio positively correlated with alpha power in the prefrontal, frontal, C3, and occipital regions, as well as with the beta power in the left prefrontal, left frontal, right frontal, parietal and occipital regions. These results suggest the existence of a negative association between estradiol action and the activation of the mirror neurons system in humans. According to the literature, the function of mirror neurons is positively associated with alpha and beta ERD in these regions during AO practice (Hari et al., 1998; Zhu et al., 2013), and in this study alpha and beta ERD decrease during AO as a function of increased estradiol level and progesterone/estradiol ratio.

Three limitations must be mentioned in this study. The first refers to the absence of a psychometric instrument to assess the vividness of MI after its execution in each experimental session. One of the reasons for this is the lack of instruments validated for Brazilian Portuguese at the beginning of data collection. The second limitation concerns the fact that participants were instructed to observe the motor action in the AO protocol, but we did not use any strategy that would ensure that their attention was maintained throughout the protocol. Thus, we recommend that in future studies participants execute or verbally report the instructed action at the end of the trials. The third one refers to the use of black and white backgrounds in the MI and AO protocol. For future studies, we suggest adopting backgrounds of the same color, which would minimize the possible effects of changes in luminosity.

## Conclusion

Although there is evidence about modulation in the neural circuitry involved in motor control throughout the menstrual cycle, there is a lack of neurophysiological studies which investigate the effects of FSS in motor cognition and executing motor actions. MI and AO are special conditions for investigating motor cognition. Our study used these two protocols to study the electroencephalographic profile of these events and the results provide evidence of beta-mu ERD on the left motor region (contralateral to the imagined action) and beta ERD modulation on the frontal and parietal regions during the practice of motor imagery according to the menstrual cycle phase. These results suggest facilitation of the pathways involved in generating beta oscillations over this region during the follicular phase when estradiol levels are high, acting in the facilitation of the motor processing. The beta ERD decrease in the medial parietal region during the luteal phase may be associated with a reduction in the vividness of motor images, but this could not be evaluated in this study. We recommend that the vividness of motor imagery in the different menstrual cycle phases be evaluated in future studies to test this inference.

No changes in menstrual cycle phases were observed in alpha ERD during MI practice nor in alpha ERD and beta-ERD during AO practice. However, it was possible to observe a positive correlation between the alpha and beta activity amplitude and the estradiol levels in the prefrontal, frontal, parietal, and occipital regions, suggesting a direct or indirect inhibitory effect of estradiol on the mirror neuron system activity.

This study shows for the first time in the literature (as far as we know) that motor cognition changes throughout the menstrual cycle, bringing evidence that MI can be facilitated during the follicular phase of the menstrual cycle, while AO does not appear to be favored by high estradiol or progesterone levels in any phase. These findings should be considered when using MI and AO to improve motor performance or in motor rehabilitation of women. This study can be used as a basis for future investigations toward understanding the potential effects of FSS on motor learning.

## Data Availability Statement

The data in this article are part of a larger study. The generated and analyzed dataset can be made available through a formal data-sharing agreement.

## Ethics Statement

The studies involving human participants were reviewed and approved by the Comitê de Ética em Pesquisa da Universidade Federal do Rio Grande do Norte. The patients/participants provided their written informed consent to participate in this study. Written informed consent was obtained from the individual(s) for the publication of any potentially identifiable images or data included in this article.

## Author Contributions

RS: conceptualization, methodology, software, validation, visualization, formal analysis, investigation, writing—original draft, writing—review and editing, and data curation. LL and TM: investigation. DB: methodology, software, validation, writing—review and editing, and data curation. DL: writing—review and editing, and supervision. MS: conceptualization, methodology, writing—review and editing, and supervision. All authors contributed to the article and approved the submitted version.

## Conflict of Interest

The authors declare that the research was conducted in the absence of any commercial or financial relationships that could be construed as a potential conflict of interest.

## Publisher’s Note

All claims expressed in this article are solely those of the authors and do not necessarily represent those of their affiliated organizations, or those of the publisher, the editors and the reviewers. Any product that may be evaluated in this article, or claim that may be made by its manufacturer, is not guaranteed or endorsed by the publisher.
