# Supplementary material for: Effect of the Menstrual Cycle on Electroencephalogram Alpha and Beta Bands During Motor Imagery and Action Observation
Source: Front Hum Neurosci. 2022 May 4;16:878887. doi: 10.3389/fnhum.2022.878887 (PMC9119141; doi:10.3389/fnhum.2022.878887)
Supplement: Supplementary file 1 [file Data_Sheet_1.pdf]

Supplementary Material

1 Supplementary material from the results

1.1 Motor imagery

Table 1. Comparation between alpha ERSP and baseline for MI

Region	Phase	Mean (dB)	SE	Z	p	p-FDR	r
**C3**	Menstrual	**-0.644**	**0.177**	**-3.178****	**0.001**	**-**	**0.410**
	Follicular	**-0.776**	**0.237**	**-2.684****	**0.007**	**-**	**0.347**
	Luteal	**-0.541**	**0.179**	**-2.787****	**0.005**	**-**	**0.360**
**C4**	Menstrual	**-0.537**	**0.216**	**-2.314***	**0.021**	**-**	**0.299**
	Follicular	**-0.602**	**0.251**	**-2.108***	**0.035**	**-**	**0.272**
	Luteal	-0.207	0.187	-0.956	0.339	-	0.123
**Left prefrontal**	Menstrual	0.121	0.218	0.566	0.572	0.910	0.073
	Follicular	0.004	0.165	-0.278	0.781	0.868	0.036
	Luteal	0.186	0.157	0.998	0.318	0.637	0.129
**Right prefrontal**	Menstrual	-0.008	0.215	0.134	0.894	0.910	0.017
	Follicular	-0.081	0.175	-0.607	0.544	0.777	0.078
	Luteal	0.152	0.172	0.730	0.465	0.663	0.094
**Left frontal**	Menstrual	-0.025	0.186	-0.113	0.910	0.910	0.015
	Follicular	-0.041	0.168	-0.710	0.478	0.777	0.092
	Luteal	0.167	0.154	0.812	0.417	0.663	0.105
**Medial frontal**	Menstrual	-0.240	0.172	-1.018	0.309	0.772	0.131
	Follicular	-0.253	0.140	-1.759	0.079	0.393	0.227
	Luteal	-0.019	0.145	-0.627	0.530	0.663	0.081
**Right frontal**	Menstrual	-0.167	0.186	-0.710	0.478	0.910	0.092
	Follicular	-0.244	0.150	-1.491	0.136	0.445	0.193
	Luteal	0.060	0.163	0.154	0.877	0.877	0.020
**Medial central**	Menstrual	-0.272	0.172	-1.306	0.192	0.737	0.169
	Follicular	-0.280	0.169	-1.347	0.178	0.445	0.174
	Luteal	0.093	0.144	0.504	0.614	0.683	0.065
**Left parietal**	Menstrual	0.063	0.224	0.381	0.704	0.910	0.049
	Follicular	-0.021	0.202	0.134	0.894	0.894	0.017
	Luteal	0.318	0.180	1.450	0.147	0.368	0.187
**Medial parietal**	Menstrual	0.087	0.259	0.257	0.797	0.910	0.033
	Follicular	0.067	0.220	0.360	0.719	0.868	0.046

	Luteal	0.453	0.213	1.944	0.052	0.173	0.251
**Right parietal**	Menstrual	0.321	0.214	1.224	0.221	0.737	0.158
	Follicular	0.252	0.197	0.895	0.371	0.742	0.116
	Luteal	0.561	0.198	2.273	0.023	0.115	0.293
**Occipital**	Menstrual	0.610	0.236	2.088	0.037	0.368	0.270
	Follicular	**0.573**	**0.179**	**2.910***	**0.004**	**0.036**	**0.376**
	Luteal	0.726	0.202	2.787	0.005	0.053	0.360

P-value: < 0.001 (***). < 0.01 (**). < 0.05 (*); FDR correction.

Table 2. Comparation between beta ERSP and baseline for MI

Region	Phase	Mean (dB)	SE	Z	p	p-FDR	r
**C3**	Menstrual	**-0.644**	**0.177**	**-3.178**	**0.001**	**-**	**0.410**
	Follicular	**-0.776**	**0.237**	**-2.684**	**0.007**	**-**	**0.347**
	Luteal	**-0.541**	**0.179**	**-2.787**	**0.005**	**-**	**0.360**
**C4**	Menstrual	**-0.537**	**0.216**	**-2.314**	**0.021**	**-**	**0.299**
	Follicular	**-0.602**	**0.251**	**-2.108**	**0.035**	**-**	**0.272**
	Luteal	**-0.207**	**0.187**	**-0.956**	**0.339**	**-**	**0.123**
**Left prefrontal**	Menstrual	**-0.324**	**0.066**	**-3.754***	**<0.001**	**<0.001**	**0.485**
	Follicular	**-0.449**	**0.074**	**-4.206***	**<0.001**	**<0.001**	**0.543**
	Luteal	**-0.261**	**0.063**	**-3.363***	**0.001**	**0.001**	**0.434**
**Right prefrontal**	Menstrual	**-0.366**	**0.074**	**-3.774***	**<0.001**	**<0.001**	**0.487**
	Follicular	**-0.491**	**0.078**	**-4.268***	**<0.001**	**<0.001**	**0.551**
	Luteal	**-0.309**	**0.070**	**-3.569***	**<0.001**	**0.001**	**0.461**
**Left frontal**	Menstrual	**-0.472**	**0.083**	**-4.165***	**<0.001**	**<0.001**	**0.538**
	Follicular	**-0.631**	**0.080**	**-4.638***	**<0.001**	**<0.001**	**0.599**
	Luteal	**-0.387**	**0.064**	**-4.062***	**<0.001**	**<0.001**	**0.524**
**Medial frontal**	Menstrual	**-0.604**	**0.083**	**-4.494***	**<0.001**	**<0.001**	**0.580**
	Follicular	**-0.748**	**0.099**	**-4.720***	**<0.001**	**<0.001**	**0.609**
	Luteal	**-0.545**	**0.069**	**-4.700***	**<0.001**	**<0.001**	**0.607**
**Right frontal**	Menstrual	**-0.483**	**0.080**	**-4.206***	**<0.001**	**<0.001**	**0.543**
	Follicular	**-0.578**	**0.072**	**-4.535***	**<0.001**	**<0.001**	**0.586**
	Luteal	**-0.426**	**0.073**	**-4.021***	**<0.001**	**<0.001**	**0.519**
**Medial central**	Menstrual	**-0.526**	**0.107**	**-3.898***	**<0.001**	**<0.001**	**0.503**
	Follicular	**-0.642**	**0.108**	**-4.638***	**<0.001**	**<0.001**	**0.599**
	Luteal	**-0.420**	**0.100**	**-3.527***	**<0.001**	**0.001**	**0.455**
**Left parietal**	Menstrual	**-0.237**	**0.091**	**-2.293***	**0.022**	**0.027**	**0.296**
	Follicular	**-0.243**	**0.097**	**-2.252***	**0.024**	**0.030**	**0.291**
	Luteal	-0.091	0.090	-1.100	0.271	0.387	0.142
**Medial parietal**	Menstrual	**-0.309**	**0.084**	**-3.075***	**0.002**	**0.003**	**0.397**
	Follicular	**-0.359**	**0.081**	**-3.754***	**<0.001**	**<0.001**	**0.485**
	Luteal	-0.092	0.096	-0.854	0.393	0.437	0.110

**Right parietal**	Menstrual	-0.093	0.089	-1.039	0.299	0.332	0.134
	Follicular	-0.190	0.084	-1.923	0.054	0.061	0.248
	Luteal	0.011	0.099	0.093	0.926	0.926	0.012
**Occipital**	Menstrual	0.001	0.097	0.154	0.877	0.877	0.020
	Follicular	-0.064	0.091	-0.257	0.797	0.797	0.033
	Luteal	0.060	0.094	0.998	0.318	0.398	0.129

P-value: < 0.001 (***). < 0.01 (**). < 0.05 (*); FDR correction.

Table 3. Effect of menstrual cycle phases in alpha ERSP for MI

Region	χ2	df	p	p-FDR	W	post hoc
**C3**	2.600	2	0.272	-	0.043	
**C4**	1.267	2	0.531	-	0.021	
**Left prefrontal**	0.200	2	0.905	0.905	0.003	
**Right prefrontal**	0.467	2	0.792	0.905	0.008	
**Left frontal**	1.667	2	0.435	0.828	0.028	
**Medial frontal**	0.200	2	0.905	0.905	0.003	
**Right frontal**	4.067	2	0.131	0.436	0.068	
**Medial central**	**11.667***	**2**	**0.003**	**0.029**	**0.194**	luteal < follicular<br>luteal < menstrual
**Left parietal**	2.467	2	0.291	0.728	0.041	
**Medial parietal**	6.467	2	0.039	0.197	0.108	
**Right parietal**	1.067	2	0.587	0.838	0.018	
**Occipital**	1.400	2	0.497	0.828	0.023	

P-value: < 0.001 (***), < 0.01 (**), < 0.05 (*), > 0.05 (ns); post hoc with Bonferroni correction.

Table 4. Effect of menstrual cycle phases on beta ERSP in MI

Region	χ2	df	p	p-FDR	W	post hoc
**C3**	8.467	2	0.014	-	0.141	folicular > menstrual<br>folicular > luteal
**C4**	2.467	2	0.291	-	0.041	
**Left prefrontal**	5.267	2	0.072	0.132	0.088	
**Right prefrontal**	5.067	2	0.079	0.132	0.084	
**Left frontal**	**11.400***	**2**	**0.003**	**0.017**	**0.190**	follicular > menstrual<br>follicular > luteal
**Medial frontal**	4.067	2	0.131	0.187	0.068	
**Right frontal**	**8.600**	**2**	**0.014**	**0.045**	**0.143**	follicular > menstrual<br>folicular > luteal
**Medial central**	2.867	2	0.239	0.298	0.048	
**Left parietal**	2.600	2	0.273	0.303	0.043	
**Medial parietal**	**12.867***	**2**	**0.002**	**0.016**	**0.214**	luteal < follicular<br>luteal < menstrual
**Right parietal**	5.267	2	0.072	0.132	0.088	
**Occipital**	0.467	2	0.792	0.792	0.008	

P-value: < 0.001 (***), < 0.01 (**), < 0.05 (*), > 0.05 (ns); post hoc with Bonferroni correction.

Table 5. Correlation between alpha ERSP in MI and hormonal level

Regions	Estradiol			Progesterone			Progesterone/Estradiol		
	rho	p-val	p-FDR	rho	p-val	p-FDR	rho	p-val	p-FDR
**Menstrual phase**									
**C3**	-0.24	0.20	-	-0.20	0.29	-	0.03	0.90	-
**C4**	0.16	0.41	-	0.03	0.86	-	-0.14	0.46	-
**Left prefrontal**	-0.17	0.36	0.44	-0.10	0.59	0.98	-0.003	0.99	0.99
**Right prefrontal**	-0.21	0.26	0.44	-0.05	0.80	0.98	0.07	0.70	0.99
**Left frontal**	-0.16	0.41	0.46	0.00	0.98	0.98	0.06	0.77	0.99
**Medial frontal**	-0.20	0.28	0.44	-0.09	0.65	0.98	0.05	0.79	0.99
**Right frontal**	-0.20	0.28	0.44	-0.03	0.89	0.98	0.08	0.69	0.99
**Medial central**	-0.06	0.76	0.76	0.09	0.63	0.98	0.08	0.67	0.99
**Left parietal**	-0.19	0.32	0.44	-0.07	0.71	0.98	0.03	0.86	0.99
**Medial parietal**	-0.20	0.28	0.44	0.09	0.63	0.98	0.17	0.37	0.99
**Right parietal**	-0.20	0.28	0.44	-0.04	0.82	0.98	0.02	0.92	0.99
**Occipital**	-0.27	0.15	0.44	-0.09	0.65	0.98	0.05	0.80	0.99
**Follicular phase**									
**C3**	0.02	0.93	-	-0.11	0.58	-	-0.06	0.74	-
**C4**	0.07	0.73	-	0.02	0.93	-	-0.02	0.90	-
**Left prefrontal**	-0.04	0.83	0.95	-0.03	0.86	0.93	0.06	0.75	1.00
**Right prefrontal**	-0.01	0.95	0.95	-0.12	0.54	0.93	-0.03	0.87	1.00
**Left frontal**	0.13	0.48	0.95	0.09	0.63	0.93	-0.04	0.83	1.00
**Medial frontal**	0.16	0.39	0.95	-0.17	0.38	0.93	-0.16	0.40	1.00
**Right frontal**	-0.03	0.89	0.95	-0.10	0.61	0.93	0.01	0.95	1.00
**Medial central**	0.04	0.85	0.95	-0.02	0.93	0.93	0.00	1.00	1.00
**Left parietal**	-0.03	0.88	0.95	-0.16	0.40	0.93	-0.02	0.90	1.00
**Medial parietal**	0.04	0.83	0.95	0.02	0.91	0.93	0.01	0.95	1.00
**Right parietal**	0.17	0.38	0.95	0.17	0.37	0.93	-0.03	0.86	1.00
**Occipital**	-0.01	0.95	0.95	0.07	0.70	0.93	0.08	0.67	1.00
**Luteal phase**									
**C3**	-0.09	0.64	-	-0.31	0.10	-	-0.17	0.37	-
**C4**	0.02	0.90	-	0.09	0.63	-	-0.01	0.95	-
**Left prefrontal**	-0.25	0.18	0.21	-0.12	0.53	0.67	-0.03	0.86	0.99
**Right prefrontal**	-0.34	0.07	0.17	-0.26	0.16	0.44	-0.09	0.63	0.99
**Left frontal**	-0.25	0.19	0.21	-0.08	0.67	0.75	-0.02	0.91	0.99
**Medial frontal**	-0.25	0.19	0.21	-0.31	0.10	0.44	-0.19	0.30	0.99
**Right frontal**	-0.28	0.13	0.21	-0.25	0.18	0.44	-0.11	0.55	0.99
**Medial central**	-0.19	0.31	0.31	-0.19	0.32	0.54	-0.10	0.59	0.99
**Left parietal**	-0.34	0.06	0.17	-0.25	0.19	0.44	-0.04	0.83	0.99
**Medial parietal**	-0.37	0.05	0.17	-0.15	0.44	0.62	0.04	0.85	0.99
**Right parietal**	-0.28	0.13	0.21	-0.05	0.80	0.80	0.05	0.78	0.99
**Occipital**	-0.35	0.06	0.17	-0.23	0.22	0.44	0.002	0.99	0.99

Table 6. Correlation between beta ERSP in MI and hormonal level

Regions	Estradiol			*Progesterone*			*Progesterone / Estradiol*		
	rho	p-val	p- FDR	rho	p-val	p- FDR	rho	p-val	p- FDR
**Menstrual phase**									
**C3**	0.02	0.91	-	0.09	0.63	-	0.14	0.45	-
**C4**	0.23	0.23	-	0.13	0.50	-	-0.07	0.71	-
**Left prefrontal**	-0.01	0.97	0.97	0.03	0.89	0.96	0.03	0.87	0.87
**Right prefrontal**	0.14	0.46	0.97	0.01	0.96	0.96	-0.06	0.76	0.87
**Left frontal**	0.09	0.64	0.97	-0.04	0.85	0.96	-0.05	0.79	0.87
**Medial frontal**	0.30	0.10	0.79	-0.05	0.79	0.96	-0.16	0.39	0.87
**Right frontal**	0.20	0.29	0.96	0.03	0.89	0.96	-0.06	0.77	0.87
**Medial central**	0.26	0.16	0.79	-0.04	0.83	0.96	-0.21	0.26	0.87
**Left parietal**	-0.01	0.95	0.97	-0.09	0.63	0.96	-0.08	0.68	0.87
**Medial parietal**	-0.06	0.77	0.97	0.05	0.81	0.96	0.05	0.78	0.87
**Right parietal**	0.02	0.91	0.97	-0.02	0.90	0.96	-0.09	0.64	0.87
**Occipital**	-0.04	0.85	0.97	-0.22	0.24	0.96	-0.19	0.31	0.87
**Follicular phase**									
**C3**	-0.18	0.34	-	-0.19	0.33	-	0.12	0.52	-
**C4**	0.28	0.13	-	-0.14	0.45	-	-0.22	0.24	-
**Left prefrontal**	-0.21	0.28	0.97	-0.31	0.09	0.43	0.15	0.44	0.97
**Right prefrontal**	-0.07	0.71	0.97	-0.11	0.56	0.62	0.08	0.68	0.97
**Left frontal**	-0.14	0.47	0.97	-0.18	0.34	0.48	0.11	0.55	0.97
**Medial frontal**	-0.01	0.96	0.97	-0.21	0.26	0.44	-0.03	0.88	0.97
**Right frontal**	-0.07	0.72	0.97	-0.08	0.68	0.68	0.13	0.48	0.97
**Medial central**	0.08	0.68	0.97	-0.33	0.08	0.43	-0.12	0.52	0.97
**Left parietal**	-0.05	0.81	0.97	-0.23	0.21	0.43	0.01	0.97	0.97
**Medial parietal**	0.10	0.59	0.97	-0.28	0.14	0.43	-0.13	0.51	0.97
**Right parietal**	-0.01	0.97	0.97	-0.15	0.43	0.54	0.03	0.89	0.97
**Occipital**	0.01	0.96	0.97	-0.25	0.18	0.43	-0.02	0.93	0.97
**Luteal phase**									
**C3**	-0.19	0.32	-	-0.25	0.18	-	-0.15	0.44	-
**C4**	0.02	0.91	-	-0.04	0.85	-	-0.20	0.28	-
**Left prefrontal**	-0.19	0.32	0.42	-0.24	0.20	0.68	-0.17	0.36	0.97
**Right prefrontal**	-0.31	0.10	0.24	-0.10	0.59	0.73	0.01	0.97	0.97
**Left frontal**	-0.18	0.34	0.42	-0.24	0.20	0.68	-0.17	0.36	0.97
**Medial frontal**	-0.14	0.47	0.52	-0.08	0.67	0.73	-0.07	0.71	0.97
**Right frontal**	-0.21	0.26	0.42	-0.07	0.73	0.73	-0.03	0.88	0.97
**Medial central**	-0.06	0.75	0.75	0.09	0.62	0.73	-0.08	0.68	0.97
**Left parietal**	-0.45	0.01	0.14	-0.38	0.04	0.40	-0.18	0.33	0.97
**Medial parietal**	-0.27	0.15	0.31	-0.13	0.49	0.73	-0.09	0.62	0.97
**Right parietal**	-0.40	0.03	0.15	-0.20	0.29	0.73	-0.04	0.83	0.97

Occipital	-0.34	0.07	0.23	-0.16	0.41	0.73	-0.05	0.81	0.97

Uncorrected P-value: <0.05 (#)

1.2 *Action observation*

Table 7. Comparation between alpha ERSP and baseline for AO

Region	Phase	Mean (dB)	SE	Z	p	p-FDR	r
**C3**	Menstrual	-0.040	0.206	0.072	0.943	-	0.009
	Follicular	0.228	0.142	1.656	0.098	-	0.214
	Luteal	0.050	0.159	0.195	0.845	-	0.025
**C4**	Menstrual	-0.361	0.225	-1.347	0.178	-	0.174
	Follicular	-0.255	0.147	-1.759	0.079	-	0.227
	Luteal	-0.219	0.190	-1.450	0.147	-	0.187
**Left prefrontal**	Menstrual	-0.352	0.229	-1.286	0.199	0.338	0.166
	Follicular	0.116	0.192	0.483	0.629	0.748	0.062
	Luteal	-0.117	0.214	-0.627	0.530	0.748	0.081
**Right prefrontal**	Menstrual	-0.349	0.226	-1.183	0.237	0.338	0.153
	Follicular	0.122	0.202	0.422	0.673	0.748	0.054
	Luteal	-0.101	0.209	-0.648	0.517	0.748	0.084
**Left frontal**	Menstrual	-0.344	0.231	-1.203	0.229	0.338	0.155
	Follicular	0.152	0.201	0.607	0.544	0.748	0.078
	Luteal	-0.118	0.189	-0.792	0.428	0.748	0.102
**Medial frontal**	Menstrual	-0.406	0.212	-1.676	0.094	0.338	0.216
	Follicular	-0.105	0.167	-0.668	0.504	0.748	0.086
	Luteal	-0.195	0.177	-1.224	0.221	0.737	0.158
**Right frontal**	Menstrual	-0.355	0.227	-1.203	0.229	0.338	0.155
	Follicular	-0.013	0.160	-0.154	0.877	0.877	0.020
	Luteal	-0.179	0.192	-0.936	0.349	0.748	0.121
**Medial central**	Menstrual	0.266	0.217	1.224	0.221	0.338	0.158
	Follicular	0.535	0.175	2.540	0.011	0.055	0.328
	Luteal	0.610	0.253	1.820	0.069	0.687	0.235
**Left parietal**	Menstrual	-0.047	0.313	0.154	0.877	0.894	0.020
	Follicular	0.438	0.230	1.656	0.098	0.266	0.214
	Luteal	0.249	0.292	0.422	0.673	0.748	0.054
**Medial parietal**	Menstrual	0.517	0.318	1.656	0.098	0.338	0.214
	Follicular	0.917	0.282	2.725	0.006	0.055	0.352
	Luteal	0.773	0.360	1.450	0.147	0.735	0.187
**Right parietal**	Menstrual	-0.119	0.329	-0.463	0.644	0.804	0.060
	Follicular	0.553	0.297	1.429	0.153	0.306	0.185
	Luteal	0.266	0.337	0.298	0.766	0.766	0.039
**Occipital**	Menstrual	-0.037	0.336	0.134	0.894	0.894	0.017

	Follicular	0.498	0.263	1.615	0.106	0.266	0.208
	Luteal	0.245	0.297	0.463	0.644	0.748	0.060

Table 8. Comparation between beta ERSP and baseline for AO

Region	Phase	Mean (dB)	SE	Z	p	p-FDR	r
**C3**	Menstrual	**-0.270**	**0.083**	**-3.054****	**0.002**	**-**	**0.394**
	Follicular	**-0.220**	**0.084**	**-2.355***	**0.019**	**-**	**0.304**
	Luteal	**-0.307**	**0.078**	**-3.157****	**0.002**	**-**	**0.408**
**C4**	Menstrual	**-0.495**	**0.094**	**-3.754*****	**<0.001**	**-**	**0.485**
	Follicular	**-0.412**	**0.091**	**-3.671*****	**<0.001**	**-**	**0.474**
	Luteal	**-0.448**	**0.102**	**-3.651*****	**<0.001**	**-**	**0.471**
**Left prefrontal**	Menstrual	**-0.276**	**0.070**	**-3.301****	**0.001**	**0.002**	**0.426**
	Follicular	**-0.143**	**0.046**	**-2.787***	**0.005**	**0.013**	**0.360**
	Luteal	**-0.215**	**0.060**	**-3.178****	**0.001**	**0.003**	**0.410**
**Right prefrontal**	Menstrual	**-0.371**	**0.067**	**-4.186*****	**<0.001**	**<0.001**	**0.540**
	Follicular	**-0.135**	**0.050**	**-2.335***	**0.020**	**0.033**	**0.301**
	Luteal	**-0.218**	**0.059**	**-3.137****	**0.002**	**0.003**	**0.405**
**Left frontal**	Menstrual	**-0.337**	**0.062**	**-4.083*****	**<0.001**	**<0.001**	**0.527**
	Follicular	**-0.267**	**0.058**	**-3.589****	**<0.001**	**0.001**	**0.463**
	Luteal	**-0.338**	**0.058**	**-4.247*****	**<0.001**	**<0.001**	**0.548**
**Medial frontal**	Menstrual	**-0.472**	**0.069**	**-4.535*****	**<0.001**	**<0.001**	**0.586**
	Follicular	**-0.372**	**0.061**	**-4.391*****	**<0.001**	**<0.001**	**0.567**
	Luteal	**-0.473**	**0.064**	**-4.535*****	**<0.001**	**<0.001**	**0.586**
**Right frontal**	Menstrual	**-0.418**	**0.076**	**-3.939*****	**<0.001**	**<0.001**	**0.509**
	Follicular	**-0.276**	**0.058**	**-3.857****	**<0.001**	**0.001**	**0.498**
	Luteal	**-0.304**	**0.057**	**-3.980*****	**<0.001**	**<0.001**	**0.514**
**Medial central**	Menstrual	**-0.260**	**0.077**	**-3.137****	**0.002**	**0.002**	**0.405**
	Follicular	**-0.164**	**0.075**	**-2.335***	**0.020**	**0.033**	**0.301**
	Luteal	**-0.200**	**0.089**	**-2.314***	**0.021**	**0.034**	**0.299**
**Left parietal**	Menstrual	**-0.410**	**0.111**	**-3.630****	**<0.001**	**0.001**	**0.469**
	Follicular	-0.139	0.084	-1.532	0.125	0.179	0.198
	Luteal	**-0.239**	**0.104**	**-2.232***	**0.026**	**0.037**	**0.288**
**Medial parietal**	Menstrual	-0.108	0.108	-0.689	0.491	0.491	0.089
	Follicular	-0.006	0.096	-0.134	0.894	0.894	0.017
	Luteal	-0.091	0.126	-1.347	0.178	0.178	0.174
**Right parietal**	Menstrual	**-0.289**	**0.115**	**-2.211***	**0.027**	**0.034**	**0.285**
	Follicular	-0.062	0.119	-0.998	0.318	0.398	0.129
	Luteal	-0.254	0.124	-1.923	0.054	0.068	0.248
**Occipital**	Menstrual	-0.258	0.121	-2.005	0.045	0.050	0.259
	Follicular	0.004	0.104	-0.339	0.734	0.816	0.044
	Luteal	-0.175	0.121	-1.409	0.159	0.177	0.182

P-value: < 0.001 (***). < 0.01 (**). < 0.05 (*); FDR correction.

Table 9. Menstrual cycle effect in alpha ERSP during AO

Region	χ2	df	p	p-FDR	W
**C3**	1.867	2	0.393	-	0.031
**C4**	0.800	2	0.670	-	0.013
**Left prefrontal**	5.600	2	0.061	0.203	0.093
**Right prefrontal**	4.267	2	0.118	0.245	0.071
**Left frontal**	4.200	2	0.122	0.245	0.070
**Medial frontal**	3.467	2	0.177	0.294	0.058
**Right frontal**	1.667	2	0.435	0.543	0.028
**Medial central**	1.267	2	0.531	0.590	0.021
**Left parietal**	6.200	2	0.045	0.203	0.103
**Medial parietal**	0.600	2	0.741	0.741	0.010
**Right parietal**	6.467	2	0.039	0.203	0.108
**Occipital**	2.400	2	0.301	0.430	0.040

Table 10. Menstrual cycle effect in beta ERSP during AO

Region	χ2	df	p	p-FDR	W
**C3**	1.400	2	0.497	-	0.023
**C4**	1.267	2	0.531	-	0.021
**Left prefrontal**	1.267	2	0.531	0.652	0.021
**Right prefrontal**	3.800	2	0.150	0.488	0.063
**Left frontal**	1.067	2	0.587	0.652	0.018
**Medial frontal**	2.400	2	0.301	0.502	0.040
**Right frontal**	2.400	2	0.301	0.502	0.040
**Medial central**	0.467	2	0.792	0.792	0.008
**Left parietal**	3.800	2	0.150	0.488	0.063
**Medial parietal**	1.067	2	0.587	0.652	0.018
**Right parietal**	3.267	2	0.195	0.488	0.054
**Occipital**	3.267	2	0.195	0.488	0.054

Table 11. Correlation between alpha ERSP in AO and hormonal level

Regions	Estradiol			Progesterone			Progesterone / Estradiol		
	rho	p-val	p- FDR	rho	p	p- FDR	rho	p	p- FDR
				**Menstrual phase**					
**C3**	-0.22	0.23	-	-0.33	0.07	-	-0.20	0.29	-
**C4**	-0.08	0.65	-	-0.20	0.28	-	-0.18	0.33	-
**Left prefrontal**	-0.07	0.70	0.73	-0.16	0.40	0.49	-0.13	0.48	0.69
**Right prefrontal**	-0.17	0.38	0.73	-0.14	0.47	0.49	-0.08	0.69	0.69
**Left frontal**	-0.15	0.44	0.73	-0.13	0.49	0.49	-0.08	0.69	0.69
**Medial frontal**	-0.07	0.71	0.73	-0.24	0.20	0.41	-0.18	0.34	0.68

**Right frontal**	-0.10	0.59	0.73	-0.15	0.44	0.49	-0.10	0.61	0.69
**Medial central**	-0.07	0.73	0.73	-0.29	0.12	0.41	-0.29	0.11	0.68
**Left parietal**	-0.12	0.52	0.73	-0.26	0.17	0.41	-0.19	0.32	0.68
**Medial parietal**	-0.10	0.61	0.73	-0.23	0.22	0.41	-0.22	0.23	0.68
**Right parietal**	-0.17	0.37	0.73	-0.27	0.16	0.41	-0.23	0.23	0.68
**Occipital**	-0.21	0.27	0.73	-0.22	0.25	0.41	-0.13	0.50	0.69
**Follicular phase**									
**C3**	**0.49***	**0.01**	-	-0.28	0.14	-	**-0.57****	**0.001**	-
**C4**	0.24	0.19	-	0.01	0.95	-	-0.21	0.27	-
**Left prefrontal**	**0.46***	**0.01**	**0.02**	-0.03	0.88	0.97	**-0.41***	**0.03**	**0.04**
**Right prefrontal**	**0.46***	**0.01**	**0.02**	-0.09	0.63	0.97	**-0.44***	**0.01**	**0.04**
**Left frontal**	**0.48***	**0.01**	**0.02**	-0.11	0.56	0.97	**-0.45***	**0.01**	**0.04**
**Medial frontal**	**0.56***	**0.00**	**0.02**	-0.11	0.57	0.97	**-0.51***	**0.00**	**0.04**
**Right frontal**	**0.50***	**0.01**	**0.02**	-0.06	0.74	0.97	**-0.46***	**0.01**	**0.04**
**Medial central**	0.36	0.05	0.05	-0.19	0.31	0.97	-0.35	0.06	0.08
**Left parietal**	0.36	0.05	0.05	0.08	0.68	0.97	-0.28	0.14	0.14
**Medial parietal**	0.36	0.05	0.05	-0.13	0.51	0.97	-0.32	0.09	0.10
**Right parietal**	0.37	0.04	0.05	-0.01	0.97	0.97	-0.33	0.07	0.09
**Occipital**	**0.45***	**0.01**	**0.02**	0.03	0.88	0.97	**-0.42***	**0.02**	**0.04**
**Luteal phase**									
**C3**	0.29	0.12	-	0.30	0.11	-	0.11	0.55	-
**C4**	0.18	0.33	-	0.28	0.13	-	0.13	0.48	-
**Left prefrontal**	0.26	0.17	0.30	0.42	0.02	0.08	0.15	0.42	0.87
**Right prefrontal**	0.21	0.27	0.30	0.39	0.03	0.08	0.15	0.42	0.87
**Left frontal**	0.30	0.11	0.30	0.43	0.02	0.08	0.14	0.45	0.87
**Medial frontal**	0.32	0.08	0.30	0.34	0.06	0.11	0.06	0.73	0.87
**Right frontal**	0.27	0.14	0.30	0.39	0.03	0.08	0.12	0.54	0.87
**Medial central**	0.29	0.13	0.30	0.28	0.13	0.16	0.02	0.90	0.90
**Left parietal**	0.24	0.20	0.30	0.32	0.09	0.13	0.08	0.67	0.87
**Medial parietal**	0.22	0.25	0.30	0.38	0.04	0.08	0.15	0.42	0.87
**Right parietal**	0.21	0.27	0.30	0.24	0.20	0.20	0.05	0.78	0.87
**Occipital**	0.13	0.48	0.48	0.24	0.19	0.20	0.10	0.59	0.87

P-value: < 0.001 (***). < 0.01 (**). < 0.05 (*); FDR correction.

Table 12. Correlation between beta ERSP in AO and hormonal level

Regions	Estradiol			Progesterone			Progesterone / Estradiol		
	rho	p-val	p- FDR	rho	p-val	p- FDR	rho	p-val	p- FDR
**Menstrual phase**									
**C3**	-0.17	0.37	-	-0.09	0.62	-	0.05	0.79	-
**C4**	0.01	0.94	-	-0.01	0.94	-	-0.05	0.77	-
**Left prefrontal**	0.06	0.74	0.94	0.01	0.96	0.96	0.01	0.94	0.94
**Right prefrontal**	0.01	0.94	0.94	-0.03	0.87	0.96	0.03	0.89	0.94

**Left frontal**	-0.03	0.89	0.94	-0.07	0.73	0.91	0.06	0.76	0.94
**Medial frontal**	0.10	0.60	0.94	-0.15	0.43	0.72	-0.15	0.42	0.84
**Right frontal**	0.18	0.35	0.94	-0.08	0.67	0.91	-0.10	0.61	0.94
**Medial central**	-0.02	0.92	0.94	-0.20	0.30	0.63	-0.17	0.36	0.84
**Left parietal**	-0.07	0.71	0.94	-0.19	0.32	0.63	-0.08	0.65	0.94
**Medial parietal**	0.08	0.69	0.94	-0.24	0.20	0.63	-0.24	0.19	0.84
**Right parietal**	-0.06	0.76	0.94	-0.26	0.17	0.63	-0.21	0.27	0.84
**Occipital**	-0.02	0.93	0.94	-0.25	0.19	0.63	-0.22	0.25	0.84
				**Follicular phase**					
**C3**	0.31	0.09	-	-0.24	0.20	-	-0.36	0.05	**-**
**C4**	0.33	0.07	-	-0.11	0.58	-	-0.30	0.11	**-**
**Left prefrontal**	**0.42***	**0.02**	**0.04**	-0.09	0.63	0.96	-0.34	0.06	0.09
**Right prefrontal**	0.25	0.18	0.18	0.04	0.84	0.96	-0.19	0.31	0.31
**Left frontal**	**0.45***	**0.01**	**0.04**	-0.20	0.30	0.75	-0.49	0.01	0.07
**Medial frontal**	0.29	0.12	0.13	-0.22	0.23	0.75	-0.34	0.06	0.09
**Right frontal**	**0.42***	**0.02**	**0.04**	0.05	0.80	0.96	-0.32	0.08	0.09
**Medial central**	0.30	0.10	0.13	-0.25	0.19	0.75	-0.36	0.05	0.09
**Left parietal**	**0.42***	**0.02**	**0.04**	-0.03	0.86	0.96	-0.37	0.04	0.09
**Medial parietal**	**0.43***	**0.02**	**0.04**	-0.24	0.21	0.75	-0.41	0.02	0.08
**Right parietal**	**0.40***	**0.03**	**0.04**	0.02	0.93	0.96	-0.33	0.08	0.09
**Occipital**	**0.48***	**0.01**	**0.04**	0.01	0.96	0.96	-0.42	0.02	0.08
				**Luteal phase**					
**C3**	0.12	0.53	-	**0.39**	**0.03**	-	0.23	0.22	-
**C4**	0.21	0.26	-	0.13	0.49	-	-0.13	0.48	-
**Left prefrontal**	0.01	0.95	1.00	0.21	0.27	0.30	0.17	0.36	0.73
**Right prefrontal**	-0.14	0.45	1.00	0.28	0.14	0.23	0.30	0.11	0.73
**Left frontal**	0.20	0.28	1.00	0.34	0.07	0.23	0.12	0.54	0.73
**Medial frontal**	0.06	0.76	1.00	0.19	0.32	0.32	0.08	0.66	0.73
**Right frontal**	0.04	0.82	1.00	0.30	0.11	0.23	0.13	0.48	0.73
**Medial central**	0.18	0.33	1.00	0.24	0.19	0.24	-0.01	0.95	0.95
**Left parietal**	0.07	0.73	1.00	0.26	0.16	0.23	0.09	0.65	0.73
**Medial parietal**	0.10	0.60	1.00	0.33	0.08	0.23	0.14	0.47	0.73
**Right parietal**	0.03	0.88	1.00	0.27	0.15	0.23	0.16	0.38	0.73
**Occipital**	0.001	1.00	1.00	0.28	0.13	0.23	0.20	0.28	0.73

P-value: < 0.001 (***). < 0.01 (**). < 0.05 (*); FDR correction.
